# Intraperitoneal ozone injection prevents REM sleep deprivation - induced spatial learning and memory deficits by suppressing the expression of *Sema3A *in the hippocampus in rats

**DOI:** 10.22038/IJBMS.2022.63994.14112

**Published:** 2022-08

**Authors:** Yi-Ning Yan, John P. Williams, Kun Niu, Wen-Hao Zhang, Jian-Feng Zhang, Le Shi, Jian-Xiong An

**Affiliations:** 1 School of Anesthesiology, Weifang Medical University & Department of Anesthesiology, Pain & Sleep Medicine, Affiliated Hospital of Weifang Medical University, Shandong, China; 2 Savaid Medical School, University of Chinese Academy of Sciences, Beijing 100049, China; 3 College of Life Sciences, University of Chinese Academy of Sciences, Beijing 100049, China; 4 Department of Anesthesiology, University of Pittsburgh School of Medicine, Pittsburg 15213, PA, USA; 5 Department of Anesthesiology, Pain & Sleep Medicine, Aviation General Hospital of China Medical University & Beijing Institute of Translational Medicine, Chinese Academy of Sciences, Beijing 100012, China; 6 Key Laboratory of Mental Health, Peking University Sixth Hospital, Peking University Institute of Mental Health, Ministry of Health (Peking University), National Clinical Research Center for Mental Disorders (Peking University Sixth Hospital), Beijing 100191, China

**Keywords:** Cognitive impairment, Midazolam, Ozone therapy, REM sleep deprivation, Sema3A

## Abstract

**Objective(s):**

Sleep deprivation is a common health problem in modern society and is negatively associated with deleterious effects on cognitive functions such as learning and memory ability. This study was undertaken to provide a detailed account of the effect of chronic ozone intraperitoneal injection on the deleterious effects of sleep deprivation on brain function in rats.

**Materials and Methods:**

Sleep deprivation was induced using the modified multiple platform model. The rats received REM sleep deprivation with an intraperitoneal injection of ozone or midazolam for 28 days. The effects of ozone on REM sleep deprivation-induced hippocampus-dependent learning and memory deficits were studied by the following approaches: Morris water maze (MWM) tests were used to evaluate spatial learning and memory of rats. Morphological changes in the brain were evaluated using hematoxylin and eosin (HE) staining. RNA-sequence was performed to seek a common mechanism. The expression of the targeted gene was examined by qPCR and Western blotting.

**Results:**

Ozone intraperitoneal injection reversed spatial learning and memory deficits associated with REM sleep deprivation, ameliorating pathological brain damage and down-regulating the hippocampal expression of *Sema3A* in rats after REM sleep deprivation.

**Conclusion:**

Ozone intraperitoneal injection alleviated sleep deprivation-induced spatial learning and memory deficits by protecting hippocampal neurons via down-regulation of the expression of *Sema3A* in the hippocampus.

## Introduction

Sleep deprivation is a public health epidemic that causes deleterious effects on brain function, especially cognition (1). It often occurs due to poor sleeping habits, psychiatric disturbances, or sleep disorders like insomnia, sleep apnea, and restless leg syndrome. There is a large body of study showing a strong correlation between sleep deprivation and memory impairment in animals and humans (2, 3). For instance, shortening the sleep cycle in rats 8 hr/day for 6 weeks impaired both short and long-term memory formation (4). Furthermore, a single night of sleep deprivation impaired motor procedural, implicit memory, and working memory in humans (5). 

Midazolam is a benzodiazepine that is often administered perioperatively as an anxiolytic and on a more prolonged basis for the treatment of sleep deprivation in patients (6). Benzodiazepines, the most commonly used sedative-hypnotics (7), produce a variety of unwanted side effects such as tolerance, abuse, and overdose (8). No medications are considered ideal agents for sleep disorder treatment. Novel therapies are still being explored.

Ozone is a strong oxidant that is often applied as a complementary or alternative therapeutic approach for wound care, autoimmune disorders, and organ ischemia. It was initially used in soldiers to prevent wound infections during the first world war (9). Ozone therapy is a non-pharmacological and low-cost procedure that is used to treat more than 50 pathological processes (10). Resitoglu *et al*. (11) found that ozone therapy could reduce neuronal apoptosis and improve cognitive function in a rat model of hypoxic-ischemic brain injury. Moreover, one study demonstrated a beneficial effect of ozone in decreasing the neurodegenerative changes of the cerebral cortex in aged rats (12). Our previous study has also shown that ozone ameliorates the behavioral and pathological deterioration in APP/PS1 transgenic mice as well as reducing the level of amyloid-β precursor protein (13). Recently, one study has found that a 3-month course of low-dose ozone therapy significantly elevated serum BDNF and GABA in insomnia patients and improved parameters of anxiety, depression, and sleep quality (14). Although the positive effects of ozone therapy have been extensively studied, the exact effects of ozone on sleep deprivation-induced learning and memory impairment in rats have not been investigated. Therefore, it is crucial to understand the molecular basis of the effect of ozone on sleep deprivation in the brain. In this study, we combined genomic and traditional molecular biology approaches to investigate the molecular effects of chronic ozone intraperitoneal injection on REM sleep deprivation-induced learning and memory impairment in rats’ hippocampus. 

## Materials and Methods

***Experimental animals***

All experiment protocols were followed in accordance with the National Institutes of Health Guidelines for Care and Use of Laboratory Animals and approved by the Ethics Committee of Aviation General Hospital of China Medical University Laboratory (HK2019-12-20). Efforts were made to minimize the number of animals and their suffering. Sixty male Sprague-Dawley rats at five months of age, weighing 350–400 g, were purchased from Vital River (Beijing, China) and maintained at Aviation General Hospital of China, Medical University Laboratory under standard housing conditions. Five rats were housed per plastic cage in a climate-controlled room (25 °C) on a 12-hr light/dark schedule (lights on at 07:00) with a*d libitum *access to standard rodent chow and water. Animals were accommodated in the laboratory environment for a week before the initiation of the experiment.

***Experimental design***

Sixty animals were randomly assigned into five groups (n=12): control group (control), wide platform group (WPF), sleep deprivation (SD), ozone / sleep deprivation (ozone), midazolam / sleep deprivation (MDZ). The rats in the control group were not given any treatment. The rats in the WPF group were placed in the same tank environment but allowed to rest on a wide platform to prevent falling. The animals in the SD group received sleep deprivation 16 hr/day for 4 weeks and received normal saline daily via intraperitoneal injections. The ozone and MDZ groups received sleep deprivation and were treated with ozone and midazolam, respectively once daily. Ozone and midazolam administration proceeded immediately after sleep deprivation ended. All manipulations started on the same day and continued for four consecutive weeks. 

***Drugs***

The O_2_-O_3 _mixture was generated by an ozone therapy device (OZOMED Basic; Kastner-Praxisbedarf-GmbH, Rastatt, Germany) at a concentration of 30 μg/ml. Ozone was administered intraperitoneally at a dose of 1.1 mg/kg body weight (15, 16). The midazolam (Jiangsu Nhwa Pharmaceutical Co., Ltd., Jiangsu, China) was dissolved in 0.9 % sterile saline and administered intraperitoneally at a dose of 2 mg/kg body weight (17).

***REM Sleep deprivation***

The REM sleep deprivation was instituted using the modified multiple platforms model, as previously described (18). Rats were placed in a large tank (150 cm in length × 80 cm in width × 55 cm in depth) made of plastic filled with 24 °C water. The tank contained 20 wooden platforms with a diameter of 6.5 cm, placed 10 cm apart (edge to edge), and arranged in 3 rows such that rats could move freely from one platform to another. It was filled with water up to 2 cm below the platform surface. When the rats reached the REM sleep stage, they lost muscle tone and fell into the water resulting in immediate awakening, following which they climbed onto the platform. This method produced an almost 95% decrease in REM sleep, which is similar to the methods that use electroencephalogram recording for sleep-deprived subjects (19, 20). To test the possible stresses of the tank environment, we used wide platforms with a diameter of 12 cm to allow the rats to sleep without falling into the water. After a 1-week adaptation period, rats from WPF, SD, ozone, and MDZ groups were sleep-deprived for 16 hr per day for four weeks (21, 22). Food and water were supplied into the grid placed on top of the water cage.

***Morris water maze***

Morris water maze (MWM) was carried out to test spatial learning and memory among rats (23). The maze consisted of a round tank (1.5 m in diameter) and a platform (10 cm in diameter), filled with opaque water (24–26 °C) to keep the platform hidden (water 2 cm above platform height). The maze was divided into four equal quadrants. The platform was located in the center of a quadrant. The behavior of the rats in the pool was monitored by an overhead video camera and analyzed by the software. The MWM test comprises two parts: the acquisition task and the probe task. Before sleep deprivation, rats were trained on the MWM task (Trial 1 and Test 1, days 8–12) to evaluate spatial learning and memory ability. Its purpose was to confirm that there were no prior statistically significant differences among the groups. 

We performed twice the acquisition tasks (Trial 1 and Trial 2) during the whole experiment time. Each acquisition task consisted of four trial sessions per day for four consecutive days. On each trial day, the rats in each group were placed into each of four different quadrants and allowed to swim for 60 sec to search for the hidden platform. Rats detecting the platform within this period were allowed to rest on the platform for 15 sec. If rats did not find the platform within the allotted time, they were guided to the platform by the investigator and remained there for 15 sec. The escape latency (time to reach the platform) was used to assess the spatial learning ability. The first trial day began on day 8 and Trial 1 lasted from day 8 to day 11. Before the end of the sleep deprivation period, we performed Trial 2 (days 37–40) to assess the learning ability of rats in each group.

**Figure 1:**
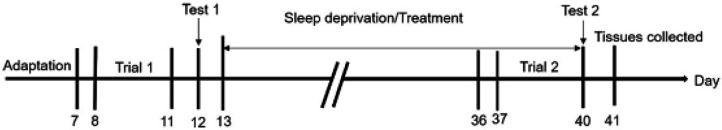
Schematic diagram of the experimental procedure

Twenty-four hours after the fourth acquisition task, the probe test was performed. The hidden platform was removed from the pool. The rats were placed at the diagonal quadrant from the original platform position and allowed to swim for 60 sec in the pool. The number of times the rat crossed over the platform site was recorded and measured as spatial memory retention. The first probe test (Test 1) was performed on day 12, which aimed to assess the memory ability of the rats in groups before sleep deprivation. Once Trial 2 finished, we performed Test 2 to assess the memory ability of rats after the experiment. The flow diagram of the animal experiment is shown in Figure 1.

***Tissue preparation***

After anesthesia with chloral hydrate at a dose of 300 mg/kg, the rats were fixed on the dissection table, and the chest cavity was fully exposed. Three rats from each group were sacrificed for detection of western blotting and quantitative PCR (qPCR). The hippocampus was removed directly after euthanasia and kept at -80 °C. For staining detection, another three rats from each group were perfused with 0.9 % saline and 4 % paraformaldehyde. The brain was removed and immersed in 4 % paraformaldehyde for 24 hr. The brain samples were embedded in paraffin, cut into 20 μm sections, and stained with HE staining. 

***Hematoxylin and Eosin (HE) staining***

HE staining was utilized to observe the neuronal morphology in rats’ prefrontal cortex, hippocampus, locus coeruleus, and amygdala in the control, SD, ozone, and MDZ groups. Following staining with hematoxylin for 30 sec, the sections were rinsed in distilled water quickly and differentiated for 6 sec in the mixed solution (hydrochloric acid 0.5 ml and 75 % alcohol 100 ml). After rinsing in distilled water for 1 hr, the sections were stained with eosin. The sections were mounted with neutral gum after dehydration through increasing concentrations of alcohol, 95 % alcohol for 2 min, 100% alcohol for 2 min, and xylene for 2 min. The sections were observed and photographed under a light microscope.

***RNA-sequence***

We extracted total RNA from the hippocampus of rats in four groups (five rats in each group) using TRIzol reagent (Invitrogen) according to the manufacturer’s protocol. The resulting total RNA was qualified and quantified using a NanoDrop and Agilent 2100 bioanalyzer (Thermo Fisher Scientific, MA, USA). After the total RNA samples were qualified, mRNA was enriched with magnetic beads containing oligo (dT) and eventually fragmented into small pieces using fragmentation buffer at a suitable temperature. Then, First-Strand Reaction System, along with polymerase chain reaction (PCR), was used for producing first- and second-strand cDNA. The cDNA fragments obtained were purified using a QiaQuick PCR kit and eluted. End repair was carried out and RNA Index Adapters were added. Finally, PCR was performed to complete library preparation. For quality control purposes, the cDNA library was validated on the Agilent 2100 bioanalyzer. qPCR was performed for accurate quantification of library effective concentration (> 2 nm) to ensure library quality. All samples were paired-ended (2 × 150 bp) and sequenced on the Illumina Hiseq2500 platform.

We filtered the raw reads to obtain high-quality reads, which were then mapped to the reference genome using HISAT2. Further, we performed differential gene expression analysis using FeatureCounts to calculate the read count value of each gene in each sample. Based on the mapping results, edge R software was then used to analyze the differential expression of genes in each sample, and the *P-*value and *P*-adjust (*P*-adj) values of that differential expression were calculated. In order to control the false discovery rate, *P*-adj values combined with fold-change were required for differential gene filtrates. Genes with a threshold of *P*-adj < 0.05 & |log2FoldChange| > 1 were screened as significantly differentially expressed genes (DEGs). 

***Quantitative PCR***

To validate the results of the RNA sequence, qPCR was carried out to examine gene expression differences in all the groups in the differential expression gene identified by the RNA sequence. The RNA concentrations were quantified using NanoDrop^TM^ 2000 spectrophotometer (Thermo Fisher Scientific, Inc.). An equal amount of RNA from each sample was reverse-transcribed to cDNA using SYBR Green qPCR Master Mix (G3322, Servicebio, Wuhan, China). qPCR was performed using CFX (Bio-rad). Primers for* Sema3A* were: forward (5’-CTTGCTCGGGACCCTTATTG-3’) and reverse (5’-AGGCTCTCTGTGACTTCGGACT-3’). Primers for GAPDH were: forward (5’-CTGGAGAAACCTGCCAAGTATG-3’) and reverse (5’-GGTGGAAGAATGGGAGTTGCT-3’). qPCR was performed with an initial denaturation step at 95 °C for 10 min, followed by 40 cycles of 95 °C for 15 sec and 60 °C for 60 sec. The relative expression of mRNA expression was analyzed with the 2^− ΔΔCt ^method, normalized to GADPH. 

***Western blot***

Hippocampal samples were lysed while on ice, with a RIPA lysis buffer containing a protease inhibitor cocktail. Protein concentration in the supernatants was measured using the BCA protein quantification kit (Servicebio, Wuhan, China). Samples were then resolved by 10 % SDS-PAGE, transferred to PVDF membranes (Merck, Billerica, Massachusetts, USA), and blocked in 5 % skim milk buffer at 37 °C for 1 hr. The blocked membranes were washed with TBST buffer three times and then incubated with primary antibodies overnight at 4 °C. Primary antibodies were obtained from the following sources: GAPDH (Servicebio, Wuhan, China, 1:1000), *Sema3A* (Santa Cruz, Shanghai, China, 1:1000). HRP-coupled goat anti-mouse IgG (Servicebio, Wuhan, China, 1:1000) was used as the secondary antibody, and protein detection was performed according to the ECL protocol. Protein quantification was conducted with Image-J software. Experiments were repeated three times.

***Statistical analysis***

All data were represented as the mean ± standard error of the mean (SEM). Significant differences between the various treatment groups for all data were determined by analysis of variances (ANOVA) followed by the Tukey *post-hoc* test. A *P*-value<0.05 was considered statistically significant for all comparisons. All statistical analyses and figures were made using GraphPad 8.0.1 (GraphPad Software, Software, San Diego, CA, USA). 

## Results

**Figure 2:**
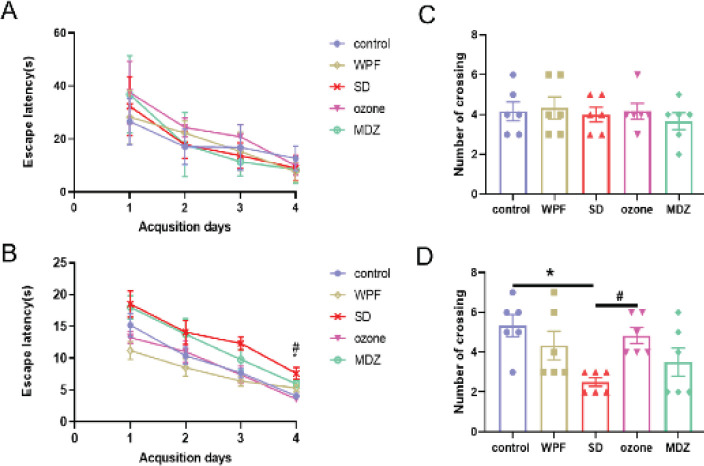
Sleep deprivation (SD)-induced spatial learning and memory impairment was prevented by ozone therapy. (A) Escape latency in trial 1. (B) Escape latency in trial 2. (C) Numbers of rats crossing through the platform in Test 1. The symbols (circles/ boxes/ up triangles/ down triangles/ diamonds) represent the individual performances in each group, respectively. (D) Numbers of rats crossing through the platform in Test 2. The symbols (circles/ boxes/ up triangles/ down triangles/ diamonds) represent the individual performances in each group, respectively. The values are mean ± SEM; *P<0.05, versus the control rat group; #*P*<0.05, versus the ozone rat group

To investigate whether ozone has a beneficial effect on spatial learning and memory ability in sleep-deprived rats, we tested spatial learning and memory using the MWM task. Acquisition tasks were conducted before and after the ozone injection. There was no significant difference in escape latency among the five groups in trial 1 (Figure 2A). While there were significant differences among each group after sleep deprivation. The escape latency in the SD group was significantly higher on the fourth day compared with the control group (*P<*0.05), indicating that REM sleep deprivation impairs the spatial memory of rats. However, the performance of the ozone group was significantly improved when compared with the SD group on the fourth day (*P<*0.05) (Figure 2B). It was also shown that the escape latencies were significantly increased in the MDZ group (*P<*0.05) when compared with the ozone group.

Then, we performed probe tests to evaluate the retention of spatial memory and recorded the number of rats crossing over the hidden platform location area. The crossing numbers before sleep deprivation did not achieve statistical significance among each group (Figure 2C). After treatment for 28 days, we practiced Test 2 to evaluate the memory. The crossing numbers in the SD group were significantly less than the control group in Test 2 (*P<*0.05). The ozone group showed increased crossing numbers with significant differences compared with the SD group (*P<*0.05). While midazolam did not improve the memory deficit compared with ozone therapy (Figure 2D). These results showed that ozone prevents sleep deprivation-induced learning and memory impairment while midazolam does not, and that performance of rats of the WPF group is similar to that of the control group.

**Figure 3:**
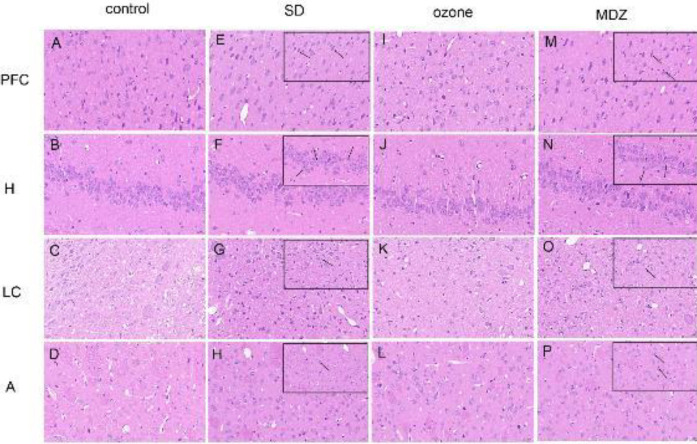
Hematoxylin-Eosin (HE) staining results. Representative images of HE staining on the prefrontal cortex (PFC), hippocampus (H), locus coeruleus (LC), and amygdala (A) in four groups. The arrow in the figure indicates the damage or the glial cell aggregation in the section. Scale bars =20 μm

HE staining demonstrated no obvious pathological change in the control group in the brain. The neurons were round and closely arranged with apparent nucleus (Figures 3A, 3B, 3C, and 3D). The neuron morphology in the ozone group was like that in the control group (Figures 3I, 3J, 3K, and 3L). In the control and ozone groups, no obvious neuronal cytoplasmic vacuolation or irregular nuclei were found. However, compared with the control group, the SD group revealed degenerated neurons with pyknotic nuclei and partially lytic forms of neuronal necrosis. It also showed an increased density of glial cells surrounding the neurons in the hippocampus and prefrontal cortex in the SD group (Figures 3E, 3F). A similar morphological change to that seen in the SD group was also apparent in the hippocampus and prefrontal cortex in the MDZ group (Figures 3M, 3N). Furthermore, the SD group and MDZ group also appeared to have neuronal injury to the locus coeruleus and amygdala (Figures 3G, 3H, 3O, and 3P), however, the damage was less than that seen in the prefrontal cortex and hippocampus. 

**Figure 4:**
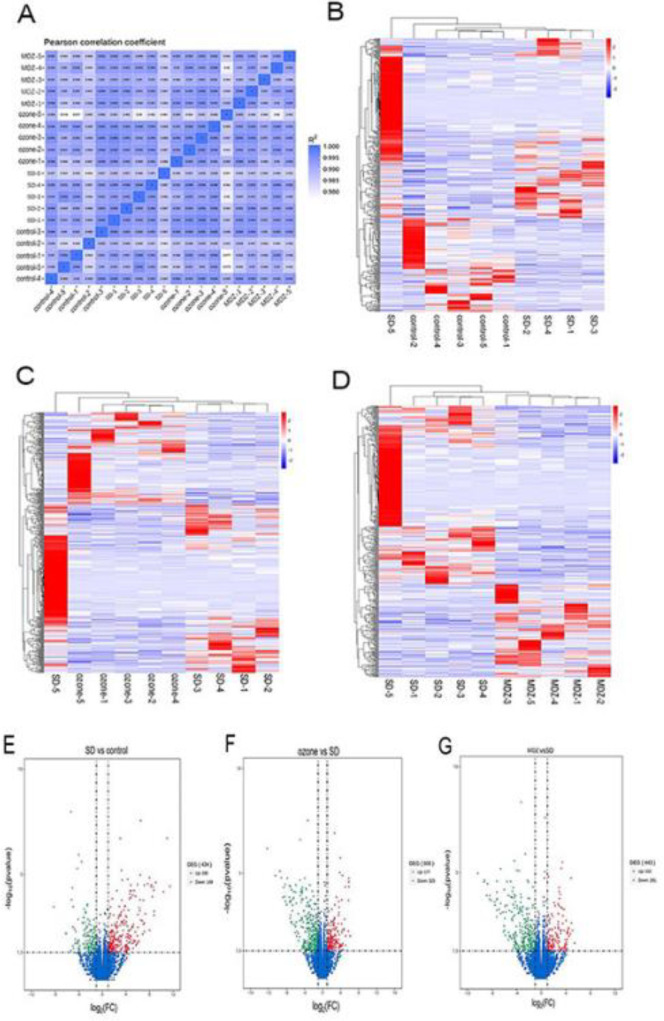
Expression of transcripts in the hippocampus. (A) Dissimilarity matrix of the RNA profile correlation clustering shows high similarity among the replicates of each group. (B/C/D) Heatmaps represent hierarchical clustering of relative enrichment (red) or depletion (blue) expressed genes from the SD group vs control group, ozone group vs SD group, and MDZ group vs SD group. (E/F/G) Volcano plot of gene expression with significantly increased (red) or decreased (green) expression (*P*<0.05, |log2| > 1) in sleep deprivation (SD), ozone, and midazolam (MDZ) groups compared with the corresponding controls

Next, we explored the gene expression changes in the hippocampus and screened DEGs among the control, SD, ozone, and MDZ groups. The RNA clustering dissimilarity matrix showed the similarity between the replicates of each group in the hippocampus (Figure 4A). To examine the molecular mechanism associated with the sleep deprivation-induced cognition deficits, we analyzed the DEGs to identify altered gene expression in the four groups following the intervention end. 285 up-regulated DEGs and 149 down-regulated DEGs were identified in the SD group relative to the control group. 177 genes were up-regulated and 323 genes were down-regulated in the ozone group relative to the SD group. Further, 152 genes were up-regulated, and 291 genes were down-regulated in the MDZ group relative to the SD group, as shown in Table 1. The relative expression heat maps of DEGs in the hippocampus were shown in Figure 4B-D. A volcano plot was used to display the distribution of DEGs in the hippocampus, as shown in Figure 4E-G, respectively.

**Figure 5:**
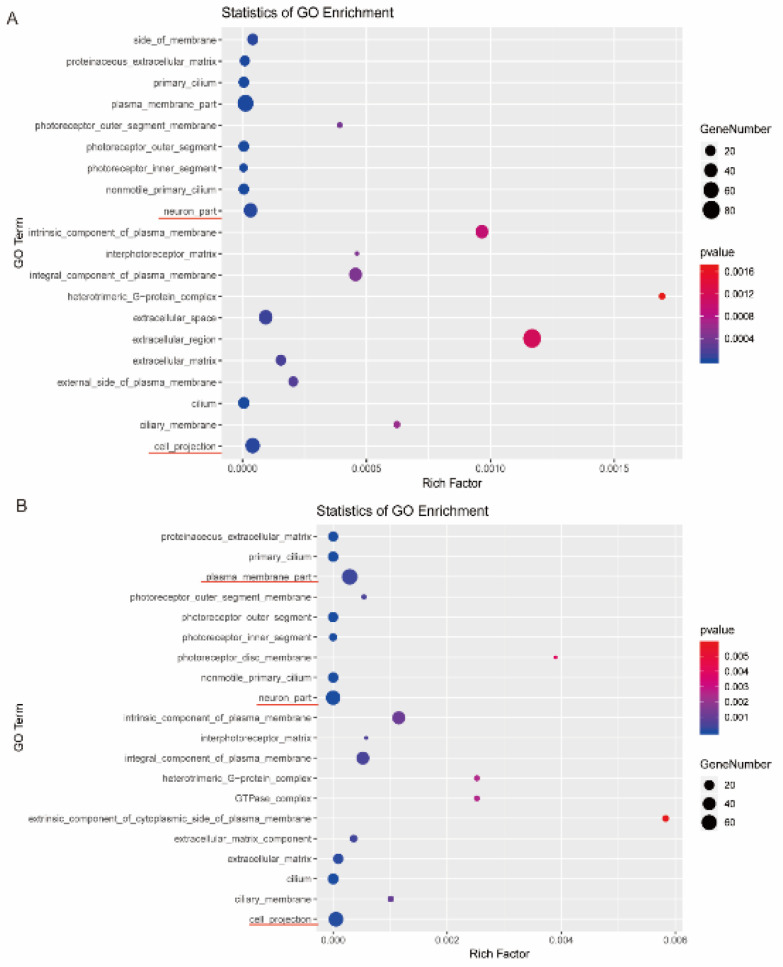
Bubble plot of the Gene Ontology (GO) analysis of DEGs. The top 20 items of potential biological function analysis were shown with parameter’s gene number and *P*-value. Each row represents a GO cellular component. The bubble plot size reflects the gene number; larger bubble plots represent more genes. (A) Top 20 up-regulated GO-Cellular Component (CC) analysis entries. (B) Top 20 down-regulated GO-CC analysis entries

To characterize the physical functions involved in these DEGs, we then performed a Gene Ontology (GO) classification and functional enrichment analysis. These DEGs were annotated in three GO domains, which are Molecular Function (MF), Biological Process (BP), and Cellular Component (CC). Figure 5A listed the top 20 up-regulated GO-CC analysis entries. The items of neuron part (GO: 0097458) and cell projection (GO: 0042995) were significantly enriched in SD vs control groups. Figure 5B listed the top 20 - GO-CC analysis entries, the items of plasma membrane part (GO:0044459), neuron part (GO: 0097458), and cell projection (GO: 0042995) enriched the top 3 gene amounts in the top 20 items in the ozone vs SD groups. 

**Figure 6:**
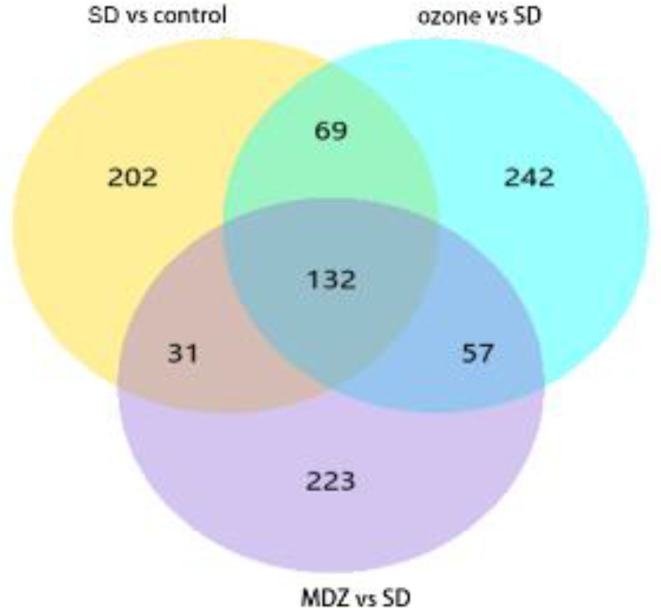
Expression of transcripts in the hippocampus in the four groups following intervention. The RNA-sequence analysis was performed for the indicated group normalized to the respective comparator group (n = 5 per group). The P-values are obtained by edge-R. The Venn diagram of differentially expressed genes (DEGs) is derived from RNA-sequencing

To examine specifically changed genes in the ozone group, we identified the expression of 69 DEGs in the SD group relative to the control group. Further, the expression of these same sets of genes resembled a more normal pattern in the ozone group (Figure 6) but not in the MDZ group. We reviewed the functions of these 69 genes in Genecards (https://www.genecards.org) and subsequently identified the cognition-related gene *Sema3A *through a literature review.

**Figure 7:**
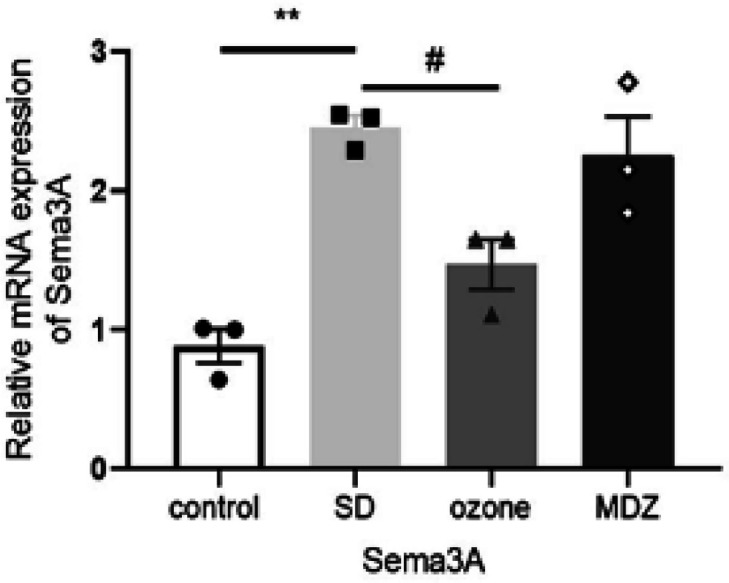
Quantitative PCR (qPCR) validation of differential expression genes (DEGs) predicted by RNA-sequencing. Results are shown as mean ± SEM (n = 3). **P<0.01: significant difference when compared with the control group; #*P*<0.05 or ##*P*<0.01: significant difference when compared with the ozone group

qPCR was conducted to verify the authenticity of the RNA sequence. *Sema3A *was selected for additional analysis by qPCR. We purposefully selected this gene as it has been documented to affect functions of the hippocampus (24, 25). Significant up-regulation occurred in the expression of *Sema3A *in the SD group compared with the control group, and the expression of *Sema3A *was down-regulated in the ozone group compared with the SD group (Figure 7). The expression of the gene examined by qPCR was consistent with that observed in the RNA sequencing results. This strongly suggests that our transcriptome sequencing results are related to our behavioral observations. 

**Figure 8:**
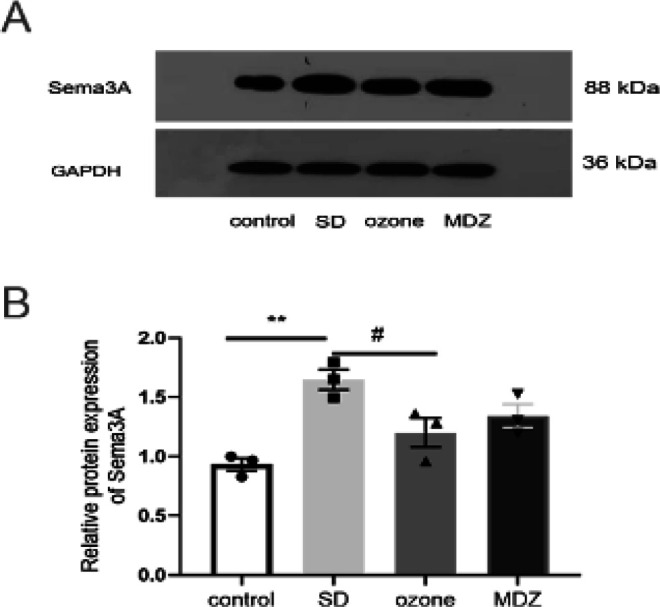
Western blot was used to observe the expression of Sema3A in the hippocampus in the four groups after 28 days of sleep deprivation. Data represent means ±SEM (n = 5). **P<0.01, versus the control rat group; #*P*<0.05, versus the ozone rat group

We analyzed the expression of *Sema3A *in rat hippocampus across the four groups. As shown in Figure 8, the expression of *Sema3A *in the ozone group was significantly decreased compared with that of the SD group. 

All rats adapted to the environment from 7 days before operation. Rats were sleep-deprived for 16h per day for four weeks (days 13-40). Rats in ozone and midazolam group received treatment from day 13 to day 40 once a day until the end of this experiment. Rats were sacrificed after Test 2 for subsequent experiments

**Table 1:** The numbers of DEGs in each comparison group

Compare	All	Up	Down
SD vs control	434	285	149
ozone vs SD	500	177	323
MDZ vs SD	443	152	291

(1) Compare: Differentially expressed genes between SD group and control group (SD vs control); Differentially expressed genes between ozone group and SD group (ozone vs SD); Differentially expressed genes between MDZ group and SD group (MDZ vs SD)

(2) All: All the differentially expressed genes

(3) Up: Up-regulated differentially expressed genes

(4) Down: Down-regulated differentially expressed genes

## Discussion

Previous work has suggested that sleep deprivation affects cognitive functions and has high comorbidity with many neurodegenerative diseases(26). We found that intraperitoneal ozone injection ameliorated the REM sleep deprivation-induced deficit of learning and memory function in rats effectively. It is similar to clinical conditions and may provide a novel basis for insomnia treatment. Moreover, transcriptome analysis revealed that down-regulation in the expression of *sema3A *may be involved in the prevention of REM sleep deprivation impairment of spatial learning and memory following intraperitoneal ozone injection. 

Multiple health risks associated with sleep deprivation lead to a reduction in one’s quality of life and some cases increase in mortality(27). In our study, we used a modified multiple platform model to perform sleep deprivation. Animals were able to move among the platforms and stayed together as a group to eliminate both the isolation and immobilization stress. The present behavioral results suggest that the cognitive ability of rats in the WPF group is not significantly different from that in the control group. This may indicate that any possible stress resulting from being in the tank may not affect the cognition impairment(28, 29). Our behavioral test data provide evidence that ozone therapy may mitigate the deleterious effects of REM sleep deprivation on spatial learning and memory ability in rats. In contrast, midazolam was not able to prevent the deficits caused by sleep deprivation. Ozone can prevent spatial learning and memory decline while midazolam cannot.

Once the behavioral experiments were completed, we observed the morphological changes of neurons in the central nervous system. We performed HE staining of the prefrontal cortex, hippocampus, and locus coeruleus amygdala of the rats. The damage caused by sleep deprivation to different regions of the nervous system, from the PFC to the amygdala, was gradually decreasing. REM sleep deprivation may probably cause more damage to higher nerve centers in the brain. One possible explanation for the results may be that the higher nerve center is more sensitive to external stimuli than the lower. Hence, we may draw an interesting conclusion that the PFC and hippocampus suffer more sleep deprivation-induced damage than the locus coeruleus and amygdala. Our results demonstrated that ozone prevents REM sleep deprivation-induced neuron injury in the brain. It would be interesting to investigate the exact mechanisms in the future. 

A current study found that low-dose ozone therapy improved sleep quality and reduced anxiety in patients with insomnia (14). Based on the study mentioned above, we hypothesized that ozone may produce a preventative effect on the cognition deficit induced by REM sleep deprivation. This study is the first to explore the effect of ozone on learning and memory impairment resulting from REM sleep deprivation.

A variety of clinical studies have demonstrated the effectiveness of ozone therapy in the treatment of degenerative neurological disorders such as multiple sclerosis, cardiovascular, and other neurological pathologies (30, 31). However, there is some debate as to whether ozone plays a positive or negative role in cognition. Ozone used to be considered a biological cytotoxic agent (32). The present knowledge allows us to understand the prolonged inhalation of ozone can be very deleterious first for the lungs and successively for the whole organism (33). Consistent with this view, ozone inhalation plays a crucial role in the pathogenesis of chronic degenerative disease (34). On the other hand, in our present experiments, we observed the beneficial effect of ozone in sleep-deprived rats. A large body of evidence has also suggested that with proper utilization, ozone is safe and effective (35). Ozone can be therapeutically used in selected diseases without any toxic or side effects (30, 36, 37). These contradictory results regarding the effect of ozone may be due to differences in the length of treatment, dosage form, and concentration. There is evidence that ozone becomes toxic at higher concentrations(10, 38), and hemolysis increasing progressively with higher ozone concentrations (39). Therefore, we used a previously documented safe and appropriate ozone concentration (30 μg/ml) for this experiment. Furthermore, intraperitoneal injection of ozone was used for treatment. 

Midazolam, a drug commonly used in anesthesiology, appears to be a useful, short-acting, sedative-anxiolytic, and amnesic prodrug. Midazolam has a fast absorption rate and is rapidly excreted after administration. As a hypnotic, midazolam is mainly indicated in insomniac patients with difficulties in falling asleep or having an abnormal sleep pattern(40). In the behavioral test, midazolam did not ameliorate the learning and memory deficits induced by REM sleep deprivation. Meanwhile, midazolam did not relieve neuronal necrosis and glia cell proliferation caused by REM sleep deprivation. These results indicated that the cognitive ability of rats kept in the MDZ group significantly decreased. Moreover, we found that some studies are in line with our findings. Midazolam administration could impair the retention of different types of memory by producing specific deleterious effects on learning or by including state-dependent memory deficits (6). We used it as a drug control to evaluate the effect on cognition in this study, when compared with ozone, even though it is widely accepted that midazolam is associated with memory impairment (41, 42). The main reason we chose it is that midazolam is widely used in anesthesiology and the treatment of insomnia. 

Due to the prominent impact of sleep deprivation on learning and memory function, we were interested in investigating the molecular mechanism of ozone on gene expression profiling in the hippocampus of sleep-deprived rats using RNA-sequence. Our funding showed that a period of REM sleep deprivation that produced deficits in cognition caused changes in hippocampus gene expression. We focused on the 69 DEGs shown in Figure 6 that have been identified as implicated in the modulation of learning and memory deficits by ozone, but not midazolam. Then, we reviewed the functions of these 69 DEGs individually. Finally, we identified the cognition-related gene *Sema3A,* which may play a significant role in the spatial learning and memory deficits associated with REM sleep deprivation. 

Semaphoring (*Sema3A*) is a secreted protein that functions in signaling growth cone collapse, maintaining structural plasticity and neuronal apoptosis during the development of the central nervous system. Previous reports have revealed that accumulation of an internalized form of *Sema3A *is associated with the degeneration of neurons in vulnerable fields of the hippocampus during AD. Accumulation of *Sema3A *overlapped the appearance of tau in many neurons, suggesting that *Sema3A *signaling at some level may be coupled to the previously identified cytoskeletal markers of neurodegeneration (43). Williams and colleagues have also shown that up-regulation of *Sema3A *in the neuronal cell bodies was corresponding to the demyelinated axons and influenced remyelination (44). Our study demonstrated that mRNA and protein expression of *Sema3A *were notably up-regulated in the hippocampus of SD group rats. This is consistent with previous findings that *Sema3A* accumulation might contribute to neurodegeneration and induce cognitive impairment. The gene we screened, *Sema3A, *may be a key target for the use of ozone in the treatment of REM sleep deprivation. In future experiments, it would be interesting to examine how *Sema3A *affects cognitive functions in sleep-deprived rats.

Our study has several limitations. First, no inhibitors or gene knockout were used, so we do not know whether ozone prevented REM sleep deprivation and caused spatial learning and memory deficits by regulating *Sema3A* protein. Secondly, we selected only one dose of ozone in this study. Further studies, specifically examining a variety of doses and concentrations of ozone are warranted. Thirdly, neurons stained with HE have only been described briefly but not statistically analyzed. In subsequent studies, Golgi staining will be arranged to count neurons to investigate the effect of ozone on the central nervous system of sleep-deprived rats. Fourthly, While midazolam is the most commonly used medication for sleep disorders, it may be more appropriate to use other medications with less cognitive impact, such as zopiclone. Finally, we did not evaluate the activity of other screened DEGs or the expression of upstream and downstream proteins in the pathways in which *Sema3A* is involved. The precise mechanism of ozone therapy still requires a great deal of further investigation.

## Conclusion

Collectively, the present findings suggest that ozone is a neuroprotective agent that prevents cognitive deficit associated with chronic REM sleep deprivation. Chronic injection of ozone prevented impairment of spatial learning and memory and protected the brain from neuroinflammation. The beneficial effect of ozone at the cellular level may be attributed to its ability to suppress the expression of *Sema3A *in the hippocampus in sleep-deprived rats. Based on our investigation, ozone can be considered a more desirable method for sleep deprivation treatment.

## Authors’ Contributions

YNY Carried out the study, performed the statistical analysis, and drafted the manuscript. JPW Helped to modify the manuscript. KN Helped to modify the manuscript and assisted in statistical analysis. WHZ Participated in behavioral and morphology studies. JFZ Helped to modify the manuscript. LS Helped to modify the manuscript. JXA Conceived the study and participated in its design and coordination. All authors read and approved the final manuscript.

## Funding

The results presented in this paper were part of a student thesis. This work was supported by the National Nature Science Foundation of China [grant number 82072086] and The Grant of Aviation General Hospital of China Medical University [grant number TB2019-011].

## Availability of Data and Materials

The datasets used and/or analyzed during the current study are available from the corresponding author upon reasonable request.

## Conflicts of Interest

The authors declare that they have no competing interests.
